# Targeting brain tumors with innovative nanocarriers: bridging the gap through the blood-brain barrier

**DOI:** 10.32604/or.2024.047278

**Published:** 2024-04-23

**Authors:** KARAN WADHWA, PAYAL CHAUHAN, SHOBHIT KUMAR, RAKESH PAHWA, RAVINDER VERMA, RAJAT GOYAL, GOVIND SINGH, ARCHANA SHARMA, NEHA RAO, DEEPAK KAUSHIK

**Affiliations:** 1 Department of Pharmaceutical Sciences, Maharshi Dayanand University, Rohtak, 124001, India; 2 Department of Pharmaceutical Technology, Meerut Institute of Engineering and Technology (MIET) NH-58, Delhi-Roorkee Highway, Meerut, 250005, India; 3 Institute of Pharmaceutical Sciences, Kurukshetra University, Kurukshetra, 136119, India; 4 Department of Pharmaceutical Sciences, Chaudhary Bansi Lal University, Bhiwani, 127021, India; 5 MM College of Pharmacy, Maharishi Markandeshwar (Deemed to be University), Mullana-Ambala, Haryana, 133207, India; 6 Delhi Pharmaceutical Sciences and Research University (DIPSAR), Delhi Pharmaceutical Sciences and Research University, New Delhi, 110017, India

**Keywords:** Glioblastoma, Brain tumor, Blood-brain barrier, Liposomes, Metallic nanoparticles, Nanocarriers

## Abstract

**Background:**

Glioblastoma multiforme (GBM) is recognized as the most lethal and most highly invasive tumor. The high likelihood of treatment failure arises from the presence of the blood-brain barrier (BBB) and stem cells around GBM, which avert the entry of chemotherapeutic drugs into the tumor mass.

**Objective:**

Recently, several researchers have designed novel nanocarrier systems like liposomes, dendrimers, metallic nanoparticles, nanodiamonds, and nanorobot approaches, allowing drugs to infiltrate the BBB more efficiently, opening up innovative avenues to prevail over therapy problems and radiation therapy.

**Methods:**

Relevant literature for this manuscript has been collected from a comprehensive and systematic search of databases, for example, PubMed, Science Direct, Google Scholar, and others, using specific keyword combinations, including “glioblastoma,” “brain tumor,” “nanocarriers,” and several others.

**Conclusion:**

This review also provides deep insights into recent advancements in nanocarrier-based formulations and technologies for GBM management. Elucidation of various scientific advances in conjunction with encouraging findings concerning the future perspectives and challenges of nanocarriers for effective brain tumor management has also been discussed.

## Introduction

Glioblastoma multiforme (GBM) is considered a belligerent form of cancer with a poor prognosis, and patients have a mean endurance of only 15–20 months regardless of maximal clinical interventions. Although it is a quite rare tumor with a global occurrence of less than 10 per 100,000 people, it has risen briskly in the previous few decades. 95% of cases were found in the supratentorial region or cerebral hemisphere, whereas few were in the spinal cord and brain stem [1]. Pathogenesis of GBM is multifaceted and engrosses alteration and mutation of numerous cellular and molecular pathways accountable for cellular survival, angiogenesis, migration, and proliferation [2]. Mutation in isocitrate dehydrogenase (IDH) enzyme is an essential pathological hallmark in the pathogenesis of GBM, and based on the mutation status of both IDH 1 and 2, GBM is subdivided into three different groups including IDH wild type, IDH mutant, and not specified [3–5]. Apart from IDH mutation, modulation in various signaling pathways, including ceramide signaling, phosphoinositide-3-kinase–protein kinase B/Akt (P13K/AKT/mTOR) pathway, notch pathways, growth factor signaling pathway, and many others, also play a decisive position in the progression of GBM [2,3].

**Figure 1:**
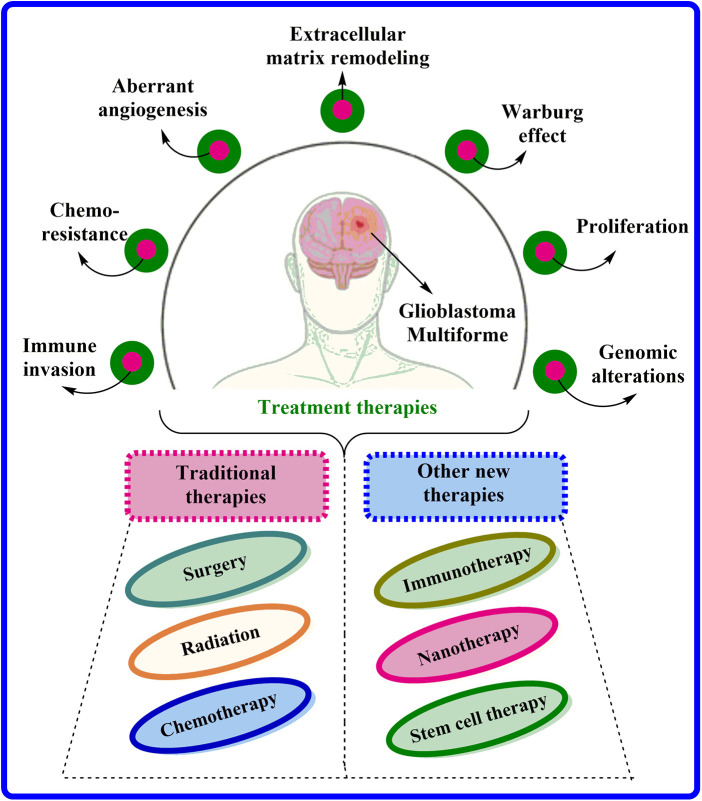
Characteristics associated with GBM and their current treatment options available.

Indeed, GBM is an aggressive and highly infiltrative ailment caused by glial cells and has limited treatment preferences available. GBM is currently treated *via* surgical tumor resection, followed by radiation and chemotherapeutic interventions [6], as illustrated in Fig. 1. An oral deoxyribonucleic acid (DNA) alkylation drug, Temozolomide (TMZ), is the primary choice for chemotherapy. TMZ is one of the few chemotherapeutic agents that can easily overcome blood-brain barrier-(BBB). Unfortunately, tumor resistance is widespread and achieved by escalating O6-methylguanine methyltransferase (MGMT) expression via demethylation of its promoter location. After surgical confiscation, biodegradable foam sheets (Gliadel® implants) can be entrenched with controlled release of carmustine, providing a slight improvement (1.1 to 3.3 months) in survival time [7]. Bevacizumab, a monoclonal antibody against vascular endothelial growth factor (VEGF), is also used to treat persistent GBM and provide symptomatic relief, primarily by reducing vascular normalization-induced edema [8]. Interestingly, tumor therapeutic field (TTF) is a novel therapeutic strategy that involves electrodes positioned on the skin to disrupt GBM cell division through low-intensity electric fields, and clinical results from a phase III trial propose that TMZ co-administration with TTF amplify overall survival from 16.0 to 20.9 months (NCT00916409) [9]. Moreover, with advancements, several newer targeted therapies are under the pipeline consisting of (Ephrin tyrosine kinase a3) EphA3 inhibitors (GLP1790, IIIA4-USAN), growth factor receptor (GFR) inhibitors (Erlotinib, Tivozanib, Sunitinib), P13K pathway inhibitors (Buparlisib, sonalisib), and many more [4,10]. Moreover, nanomedicine has gained more popularity than others because of its various benefits including less particle size, greater bioavailability, specific site targeting and several others. Researchers worldwide actively explore novel formulations to enhance brain drug delivery in this rapidly evolving field. Many challenges persist, especially in generating highly efficient, therapeutically beneficial, and practical treatments for brain cancer.

### Blood-brain barrier (BBB) as an obstacle to drug delivery

**Figure 2:**
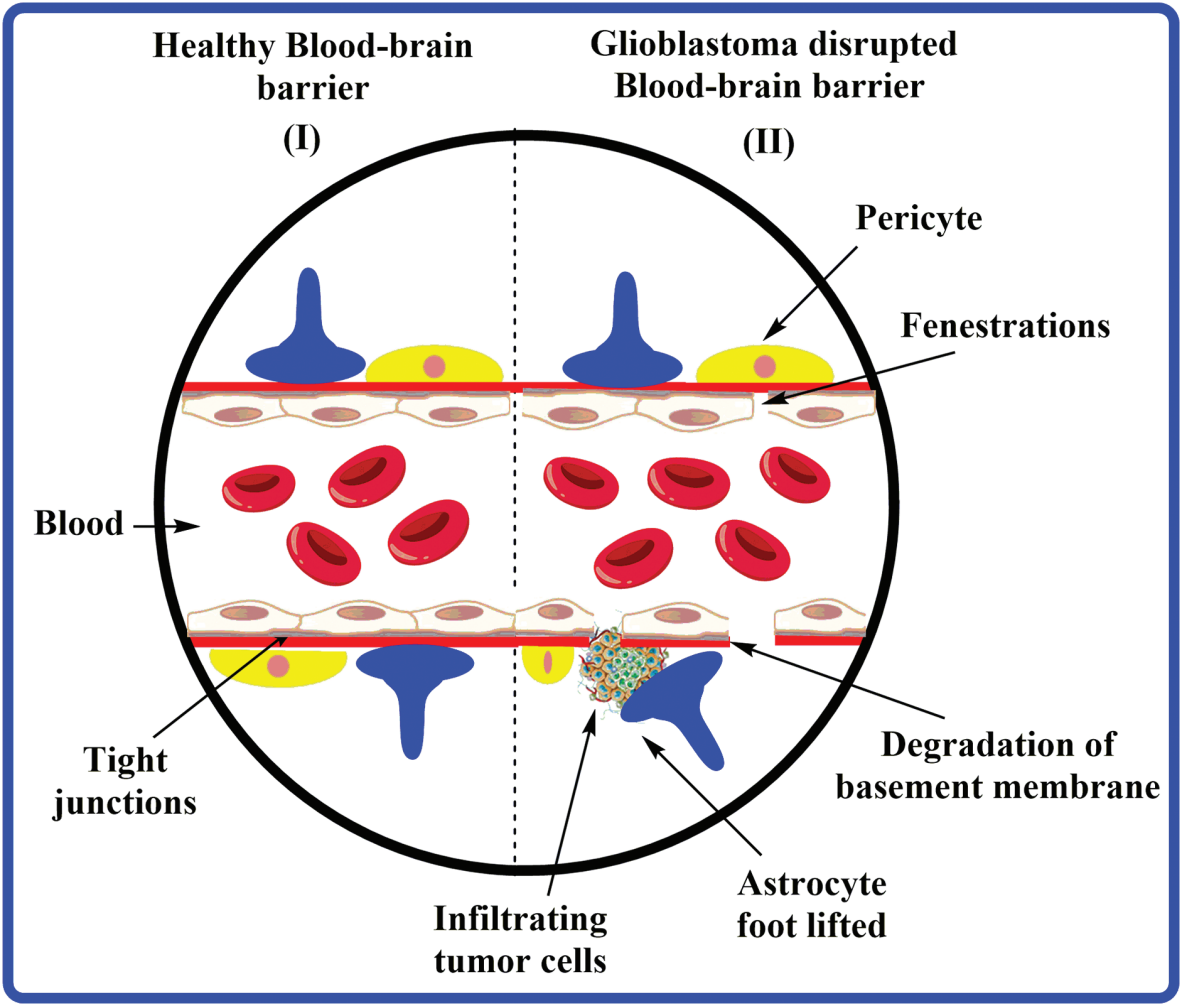
Characteristics of healthy BBB *vs.* GBM-affected BBB.

The BBB comprises brain endothelial cells (BECs) advocated by astrocytes and pericytes terminal axes. These BECs are highly specific, non-pyrogenic, and have a meager cell transfection rate [11]. In addition, BECs utilize the angiogenic agents claudin-5 and zonulachludens-1 (ZO1) to form tight junctions that prevent the cellular migration of water-soluble compounds [12,13]. These BECs, collectively with neurons, develop the neurovascular unit. Indeed, in response to any injury, the pericytes permute BEC gene expression and trigger astrocytes to liberate growth factors that have the potential to alter barrier physiology [14,15]. The BBB preserves the brain homeostasis and acts as a shield, abandoning the entry of toxins and pathogens into the brain by regulating the haulage of substances across the meninges and the abluminal (facing the brain) [16]. Despite its name, BBB is more than just a barrier; it is conceivably more correctly illustrated as an “interface” with multiple functions [17]. Fig. 2 portrays the features of healthy BBB compared to GBM-affected BBB.

Based on their physiochemical properties; drugs and endogenous compounds cross BBB via passive diffusion, endocytosis, active transport, or carrier-mediated transport. Several efflux and influx transporters, such as ATP-binding cassette (ABC) and solute carrier (SLC), present in BEC cells are primarily accountable for the uptake and extrusion of various compounds and metabolites [18,19]. Although several general rules for drug delivery to the brain have been postulated regarding molecular permeability, many exceptions still exist [20]. Unless a specific transporter, larger molecules with more than 400 Da find it complicated to pass through the BBB, mainly if they are water-soluble. Conversely, small, fat-soluble compounds are more permeable and easily penetrate BBB. Likewise, TMZ, with its molecular weight of 194.1 g/mol and low water solubility, easily overcomes BBB and is one of the few available interventions for GBM [21,22]. It should also be taken into account that lipid solubility is not only the limiting factor for the significant accumulation of drug concentration in the brain; its metabolism and clearing by efflux pumps can also alter the penetration of the drug into BBB [8,23]. High plasma protein binding of a drug also makes it non-eligible to cross BBB because it requires specific transporters for movement [24]. Overall, these limitations, combined with the features of Lipinski’s “rule of five,” proposed that most chemotherapies used today are unsuitable for brain delivery. Also, the preponderance of established anti-cancer therapies fades because of non-targeted toxicities, which can be avoided by developing target-specific drug delivery systems. Nanotechnology advancements have facilitated targeted methods to triumph over drug delivery obstruction allied with brain and other types of cancer [8]. The present review has been designed to provide insights into recent advancements in nanocarrier-based formulations and technologies for GBM management. Elucidation of various scientific advances in conjunction with encouraging findings concerning the future perspectives and challenges of nanocarriers for effective brain tumor management has also been discussed.

## Nanomedicines for Enhanced BBB Permeability

**Figure 3:**
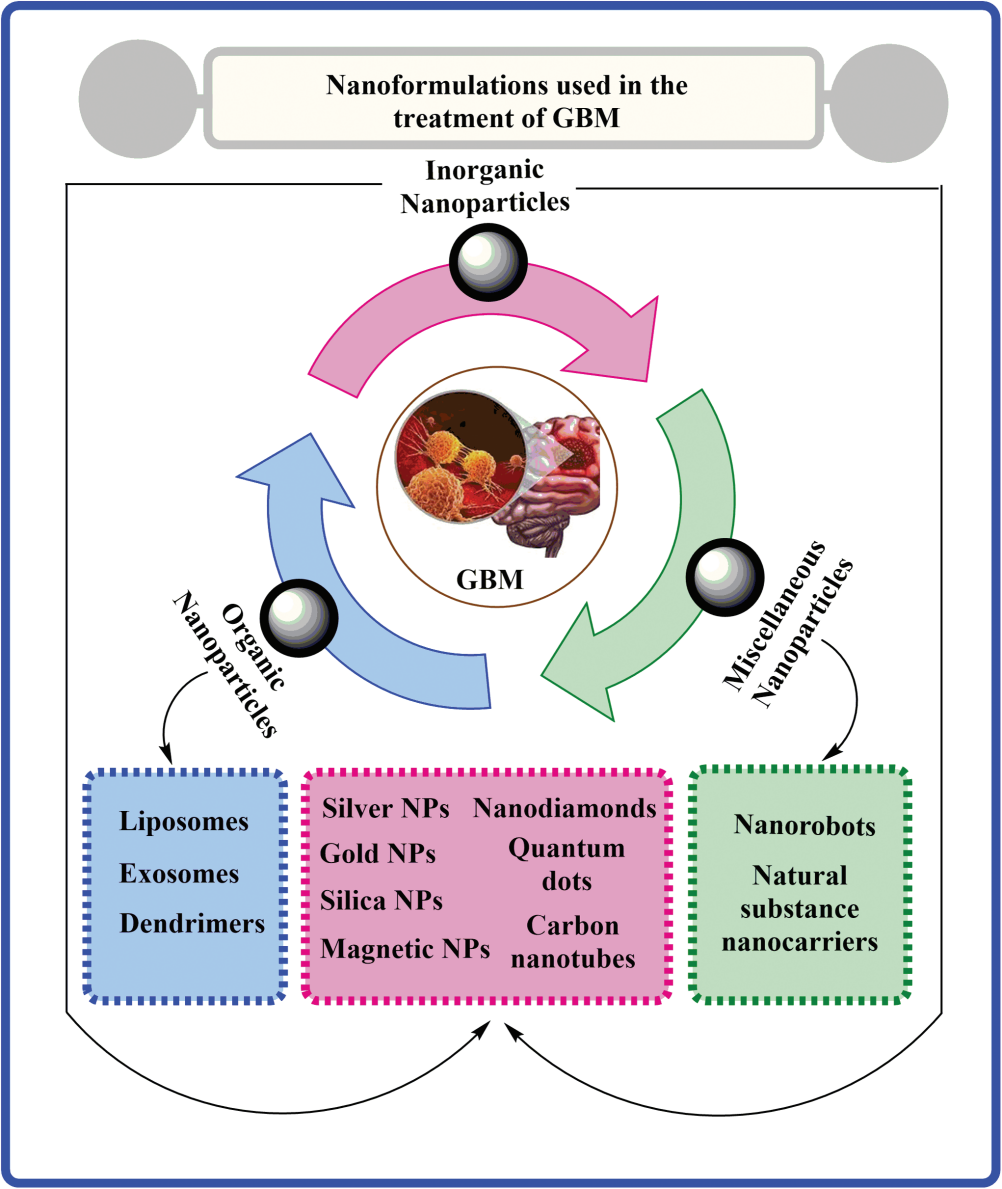
Various nanoformulations employed in GBM treatment.

Nanomedicines are small materials with diameters ranging between 1–100 nm that have numerous therapeutic or diagnostic relevances in brain cancer [25], as shown in Fig. 3. Nanomedicine formulations are considerably innovative and effective propositions for drug delivery via BBB compared to conventional drug delivery systems. These nanomedicines are readily permeable to tiny capillaries of BBB by their nanoscale size, and controlling physicochemical properties can modify the drug biodistribution and pharmacokinetics [26]. In addition to their small size, nanocarrier-based drug therapies for GBM offer crucial advantages such as a low toxicity profile, high target specificity, and controlled release, making them highly beneficial [27]. Indeed, the physicochemical characteristics of nanotechnology-based medicines are imperative factors for their action within the body [26,28]. The shape and diameter of nanomedicine, along with its surface factors like smoothness, charge, and chemistry, primarily affect the pharmacokinetic and pharmacodynamic behavior, comprising drug accumulation in the brain [29,30].

Using a human BBB model, Brown et al., investigated the influence of nanoparticle size, form, composition, and rigidity on the uptake mechanism by incorporating intracellular inhibitors cell-mediated clathrin and caveolin to distinguish the outcome obtained by each determinant on the absorption and pharmacokinetic properties. Even though there was a size-specific result, it was also observed that particle composition has been a very crucial limiting factor that should be taken into consideration. For instance, 500 nm-sized transferrin nanoparticles (NPs) penetrate BBB more efficiently than liposomal nanoparticles (NPs) of a similar or lesser size [31].

Studies also affirmed that disease conditions could alter both BBB physiology and nanomedicine delivery *in vivo* [28,32,33]. Houston and colleagues employed mice with spontaneous brain tumors in a critically vital study to underline the relationship between BBB permeability with nano drugs and tumor growth. Compared with post-contrast magnetic resonance imaging (MRI) changes showing leakage, it was observed that tumor volume solitary acts as a pathetic prophet of BBB permeation. In addition, it was also affirmed that BBB’s permeability varied according to the brain tumor’s location, indicating that this heterogeneity poses a challenge in drug delivery [34]. Numerous nano-based drug delivery systems that can effectively transport the drug to the brain by the voyage of BBB have been formulated and developed with unique targeting mechanisms and provide a better anti-tumor therapeutic effect [35,36]. The present section highlights the numerous nanocarrier systems, including liposomes and polymeric micelles, exosomes, carbon-based NPs, metallic NPs, and other biomimetic systems that are believed to be most suitable for clinical translation.

## Organic Nanocarriers

### Liposomes

The liposome is one of the most advanced nanoscale drug delivery vehicles, capable of delivering chemotherapeutics, antibiotics, analgesics, and vaccines [37]. Naturally or synthetically, liposomes are spherical hydrogel-filled vesicles made of lipids with a small aqueous center inside. Stiffness, phase transition temperature, and stability are affected by lipid composition, subsequently affecting drug encapsulation and preservation aspects [38]. In the case of lipophilic drugs, molecules can be packaged in the associated lipophilic segment of the membrane. Polyethylene glycol (PEG) binds to the outer membrane of liposomes, creating a protective layer that shields them from the reticuloendothelial system, escalating circulation time and plummeting immunogenicity [37]. Also, high-density PEG coatings generally improve brain tissue penetration [39]. PEG is usually considered safe, but complement activation and development of anti-PEG IgG and IgM antibodies in some individuals raise limitations in its effectiveness [40,41]. Amplifying circulation time and diminishing off-target accumulation using liposomes can advance the safety and usefulness of encapsulated active ingredients [42]. Interestingly, it was calculated that 100 nm NPs should contain approximately nine PEG molecules (5kDa) per 100 nm^2^ particle surface for the most evident passage through the brain’s parenchyma [39].

Liposomes are an appealing option for BBB targeting, particularly in treating GBM. Gao and associates found that encapsulating TMZ in simple liposomes with phospholipid/cholesterol augments pharmacokinetic characteristics. They also showed that brain uptake of TMZ was also enhanced compared to free drugs [43]. Nevertheless, liposomes can transport their cargo to the brain via three different mechanisms, including transporter-mediated cell transfer (TMT), adsorption-mediated cell transfer, and receptor-mediated cell transfer (RMCT), which subsequently interacts with proteins of glutathione transporter and transferrin receptor (TfR), widely found in BECs [42]. Several PEGylated liposomes encapsulating doxorubicin (DOX) have been fabricated using glutathione to improve BBB permeability by targeting GSH receptors [44,45]. Targeting TfR also promotes BBB delivery of drugs. Recent research proposed that liposomes prepared using TfR-targeting peptide not only improve drug uptake to the brain but also promote glioma-targeted drug delivery by 4-fold [46]. Also, a TfR-T12 peptide-modified PEG-PLA micelle encapsulating paclitaxel (PTX) significantly surmounts the BBB and elevates intratumoral drug delivery by 2-fold [47]. Jhaveri et al., fabricated resveratrol-loaded liposomes, and results affirmed that TfR-targeted liposomes not only prolong *in vitro* drug release and BBB penetration but also trigger cell arrest and cellular apoptosis, consequently prolonging overall survival in mice [48].

Both healthy and tumor brains have deficient uptake of PEGylated liposomes by default. A recent study using pigs affirmed that PEGylated liposomal DOX (≥0.5% IV infusion) readily crosses the BBB, whereas, in mice, less than 0.1% of the dose significantly penetrates the BBB [49]. Indeed, the tumor progresses permeability because of the chaotic and leaky vasculature, yet the blood-brain tumor barrier (BBTB) can protect against BBB leakage [50]. Noteworthy, LipoDox accumulates 2.3 times more in an orthotopic xenograft of a human GBM tumor than typical brain tissue in the murine model, indicating extremely diminutive enhanced permeability and retention (EPR) effects. Moreover, skin implantation of LipoDox elevates its accumulation by 25.8 folds, representing unequivocally that BBTB protects GBM tumors when liposomes are administered systemically [49]. However, few results showed no benefit in treating GBM patients with PEGylated liposomal DOX in conjunction with TMZ [51].

As a result, liposomes need more assistance than passive uptake to enter the brain adequately [52], which can be done by impregnating liposomes with penetrating, targeting, or shielding properties [53]. A fundamental study by Aryal et al., concluded that delivering liposomal DOX to the brain through focused ultrasound-mediated BBB disruption in healthy rats may induce neurotoxicity and cause damage to the brain [54]. Since the 1950s, ultrasound has been acknowledged to alter BBB integrity [55]. Yet, several challenges concern developing and scaling up to human skull thickness, including safety issues [56]. A small human trial conducted recently affirms the possibility of combining focused ultrasound with liposomal DOX or TMZ. The results showed improved drug concentration in the focused ultrasound-treated section of non-sonicated tumor margin tissue obtained [57]. Studies also postulated that VEGF could temporarily open the BBB, allowing PEGylated liposomes to be delivered more effectively to brain tumors [49]. Utilizing this approach, Mamot and their team formulated anti-endothelial growth factor receptor (EGFR)-DOX immunoliposomes, which showed 3.5–4.9 fold amplification in DOX accumulation in nuclei with 3.5–8 times more in the cytoplasm compared to free DOX in preclinical studies [58], which later on also found safe and tolerable in Phase 1 clinical trial on the patient with advanced solid tumor [59,60].

The common approaches to trigger drug release from liposomes involve modulating pH and temperature, as encapsulated drugs in liposomes cannot offer therapeutic effects. As hyperthermia fleetingly unwraps the BBB, Bredlau and his associates developed temperature-sensitive DOX liposomes to facilitate the localized delivery of DOX in the brain [61]. Although over 20 years of research have been conducted to triumph over precincts associated with liposomal drug release, limited clinical trials were done using the hyperthermic approach to trigger drug release because of restricted brain injury, and some animals developed side effects as a result [62–64].

Exploiting the inflammatory microenvironment also endorses glioma-targeted drug delivery, so interleukin (IL)-13 conjugated liposomes of DOX were fabricated that decreased tumor volume by 5-fold within 6 weeks in mice U87 xenograft model [65]. Similarly, IL-4 receptor-targeted DOX liposomes promote tumor-targeted delivery and improve the antitumor effect in GBM [66]. Using combination technology, Zhao and their team developed shRNA-loaded liposomes (~100 nm) that significantly uphold drug delivery by 8.5 folds and improve survival rate [67]. Yet, liposomal stability and longevity are a few limitations allied with it. Physiologically, glucose transporter (GLUT)-1 transporters are available on BECs, and targeting these hexose transporters can augment the drug’s penetration across BBB. Using this hypothesis, Anraku et al., fabricated PEGylated liposomes with multiple glucose molecules per nanoparticle. Hi-jacking glucose transporter was observed to improve drug uptake in the brain by 20-fold [68].

For drug delivery to the brain, non-targeted liposomes have also been investigated. Several clinical studies have used liposomal DOX (Myocet) for the treatment of recurrent gliomas in children (NCT02861222), and liposomal irinotecan has also been studied for the treatment of recurring high-grade GBM (NCT02022644) [69]. In 2008, a Phase I study investigated the therapeutic potential of liposomal irinotecan (NL CPT-11), a free drug that failed a Phase II trial in patients with GBM [70]. Yet, another liposomal irinotecan (MM-398) demonstrated a significant anti-tumor effect in patients with advanced breast cancer with brain metastases (NCT01770353) [71].

### Extracellular vesicles and exosomes

Cells release membrane-bound nanoscale vesicles known as extracellular vesicles (EVs). These EVs include microvesicles, exosomes, and apoptotic bodies with sizes ranging between 100–1000, 30–150 and 50–5000 nm, respectively, and they also differ in composition, function, and biogenesis [72]. EVs are prevalent in human bodily fluids and act as a bridge between cells and tissues [73]. Platelets and bacterial microbiomes can also contribute to EVs in the blood. Exosomes are generally generated by splitting out from cell membranes and contain highly complex components that vary in protein, lipid, and cholesterol concentrations [74]. In contrast to most manufactured nanomedicines, exosomes may instantly bind and engage with receptors on particular target cells [75]. Because of their tiny size, exosomes have garnered the most scientific interest among EVs since they can easily carry miRNAs, enzymes, and several peptides incorporating growth factors. Surprisingly, the composition of EV contents does not always match that of the starting cell, and protein quantity might diverge up to 100-fold [72]. Exosomes can be utilized on their own or in combination with other treatments. Exosomes are best employed for anti-inflammatory or immune-modulatory objectives because they don’t possess tumor-killing activity alone. Reducing inflammation is helpful in central nervous system (CNS) applications, including neurodegeneration and traumatic brain injury. Exosomes, conversely, can be modified to function as tumor-killing agents by changing the parent cell by overexpressing miRNAs that consequently prevent angiogenesis or cell proliferation [76]. Drugs and genes can be transported via exosomes loaded with exogenous substances. Exosomes have been developed to produce sh-RNA and si-RNA targeting oncogenic KRAS, effectively reducing metastasis and lengthening life in various pancreatic cancer mouse models [77].

Exosomes are considered a promising option for brain tumor delivery, yet have received far less attention than NPs, liposomes, and various other nano-technologies. Exosomes produced from Human embryonic kidney 293 (HEK293T) were shown to permeate BBB more effectively than liposomes or any other polymeric NPs when studied *in vitro* and showed significantly elevated therapeutic effectiveness *in vivo* [104]. According to Morad and coworkers, reduced endothelial *rab7* expression facilitates BBB permeability through transcytosis in exosomes generated by brain-seeking breast cancer cells [105]. Biologically, the surface of blood-derived EVs has transferrin-TfR complexes, which promote their absorption into the brain [106]. However, the cells that produce exosomes cross BBB differently. For instance, in several studies, exosomes from endothelial cells have been demonstrated to intermingle with BBB, but exosomes isolated from cultured GBM cells fail to do so. Exosomes generated by hypoxic GBM cells, on the other hand, were competent to modify BBB integrity [107]. Cancerous cells liberate exosomes that perturb the BBB and consequently trigger the brain for metastasis [108]. On examining exosomes obtained from 10 different cancerous and non-cancerous cell lines, it was observed that all of them can cross the BBB to some level, and the degree of brain absorption is assorted by a factor of ten [109]. Exosomes produced by macrophages may be a suitable option since they can infiltrate BBB easily and deliver brain-derived neurotrophic factor (BDNF) to the brain following IV administration [110]. Exosomes can infiltrate the BBB, so they might be employed to deliver traditional chemotherapeutic interventions. Drugs can be encapsulated into exosomes by sonication, electroporation, freeze-thaw, surfactant use, passive absorption, and simple incubation [107]. Yang et al., demonstrated that bEND.3-derived exosomes carry DOX and PTX (7.3 ng and 132.9 ng, respectively, per 1 g exosome) and outperform free drugs in a zebrafish model [111]. Exosomes made from EL-4 cells and loaded with curcumin and a Stat3 inhibitor can be easily absorbed by microglial brain cells, along with a significant reduction in the production of inflammatory cytokines and elevation in the lysis of tumor cells when administered intranasally [112].

### Dendrimers

A significant issue for the treatment of GBM is the dispersion of chemotherapeutic medications across the BBB and their effectiveness and targeting capabilities. With or without targeting ligands, the branching molecular configuration of polyamidoamine (PAMAM), poly-L-lysine (PLL), and poly-propylene-imine (PPI) dendrimers have shaped an enormous prospective for nanotechnology to aid the transport of chemotherapeutics over the BBB to manage brain tumors efficiently [113]. Targeting ligands can be added to dendrimer-mediated targeting strategies to promote interactions with particular receptors. Indeed, sugar moieties play a crucial role as ligands on hydroxyl-terminated PAMAM dendrimers for cancer immune targeting and altered metabolism. Researchers employed a relatively straightforward click chemical technique to alter the facade of dendrimers with several sugar moieties to target better sugar transporters in the milieu of GBM [114]. According to Sharma and colleagues, increasing brain penetration and cellular internalization significantly elevates tumor-associated macrophages (TAMs) and microglia targeting. In contrast, galactose modification changed the targeting of GBM cells away from TAMs and toward galectins. Although mannose modification did not affect these dendrimers’ ability to target TAMs and microglia, it impacted accumulation inside the GBM. Furthermore, to selectively transfer the BLZ945, a (Colony-Stimulating Factor 1 Receptor) CSF-1R inhibitor, in a targeted approach via repolarization of the tumor immune milieu, Sharma et al. produced a unique hydroxyl dendrimer-mediated immunotherapy. In contrast to free BLZ945, a sole systemic dosage of D-BLZ customized to TAMs reduces pro-tumor expression and boosts cytotoxic T-cell infiltration, leading to extended endurance and reduced burden [114].

Knauer et al., believed that tumor cells with stem cell characteristics are essential in fostering aggressive malignancies’ growth and malignant activity. Effectively removing these tumor stem cells is a highly required clinical need as a specific therapeutic approach [115]. The polycationic phosphorus dendrimer-based system for delivering short interfering RNAs was confirmed using an *in vitro* stem-like cell model. It was shown that anti-Lyn siRNA given into glioma cell models reduces cell viability and impacts various cell characteristics, such as the expression of the surface markers CD47 and TIM-3, immunological recognition targets, and other physiological functions that may affect glioblastoma cell invasion. Results showed that dendrimer-based platforms have therapeutic promise and may help exterminate cancer cells [115]. Liu et al., fabricated and modified a PAMAM dendrimer-based carrier incorporating angiopep-2 (Ang2) peptide that considerably ally to low-density lipoprotein receptor (LDLR)-relative protein-1 (LRP1) on the BECs and triggers BBB infiltration of the carrier. The dual-targeting dendrimer displayed stupendous BBB permeability and target specificity both *in vitro* and *in vivo*, which prominently improved the anti-cancer potential and lengthened the existence of glioma-bearing mice [83].

Therefore, dendrimer systems are among the most widely utilized nanocarrier systems. These are crucial tools for treating brain tumors due to their many properties, including physicochemical characteristics, composition, and surface functionality, that can be altered for active targeting. Dendrimers show great potential for treating brain cancers due to their capacity to deliver molecular payloads to tumor sites and their effectiveness in permeating the BBB and entering the brain [116]. Interestingly, intranasal administration enables undeviating drug deliverance into the brain without bridging the BBB. In this light, dendrimers may potentially treat CNS illnesses by circumventing the BBB and plummeting systemic contact with medications while reducing adverse effects [117].

## Inorganic Nanocarriers

### Magnetic nanoparticles (MNPs)

Although inorganic nanocarriers have spawned much research interest as therapeutic agents, their clinical applications in GBM are restricted compared to liposomes. There are frequently few treatment options available when GBM reoccurs. Maier-Hauff and associates looked into the possibility of improving survival outcomes by applying a novel intratumoral thermotherapy technique utilizing magnetic nanoparticles (MNPs). An aqueous dispersion of Fe_2_O_3_ MNPs was injected into the tumors of 66 patients (59 of whom had recurrent GBM) in a single-arm research conducted in two centers. The primary trial outcome was overall survival after the initial tumor recurrence diagnosis (OS-1), and the secondary endpoint was overall survival following the identification of the initial tumor (OS-2). Among the 59 patients with recurrent GBM, the mean overall survival from the marker of the first tumor recurrence was 13.4; in contrast, the average time amid the first tumor reappearance was 8.0 months, and the mean OS-1 was 23.2 months. The outcomes thus affirm that MNPs thermotherapy combined with a lower radiation dose is safe, effective, and extends OS-2 compared to traditional therapies [118]. The European Union approved this MNP-based thermotherapy in GBM patients based on the results obtained from clinical studies, yet the procedure has several drawbacks, including seizures and the requisition to remove all dental implants [119,120]. With exceptionally improved physicochemical properties, such as catalytic, optical, thermal, and electrical properties, MNPs have bactericidal, antiviral, fungicidal, and antioxidant potential. According to studies, these NPs are also used in a range of biological fields, including drug delivery, cancer treatment, and diagnostics [121].

Due to its low level of invasiveness, the permutation of photothermal therapy (PTT) with chemotherapy is proving to be an effective method for treating GBM. Reduced undesirable side effects and increased treatment efficacy are two critical goals in improving innovative therapeutic compounds for chemo-photothermal therapy [119,120]. Due to their low toxicity and distinctive magnetic characteristics, iron oxide NPs are frequently utilized as efficient drug carriers. Fe_3_O_4_ MNPs have recently been thoroughly proven to be efficient PTT agents for cancer therapy using near-infrared (NIR) light. However, the mechanism by which Fe_3_O_4_ MNPs induce cellular apoptosis has not been extensively analyzed, particularly the effect of an anti-GBM medicine inside cancer cells due to the photothermal effect [121]. In a pre-clinal study, Cho and associates investigated how Fe_3_O_4_ MNPs supported efficient chemo-PPT-induced cell killing in U87-MG brain cancer. To treat brain cancer, they successfully fabricated multifunctional Fe_3_O_4_ MNPs loaded with TMZ. The temperature of the synthetic Fe_3_O_4_ MNPs was noticeably raised during NIR laser irradiation at 1 W/cm^2^ for 5 min, and intracellular drug delivery was improved. Additionally, U87-MG brain cancer cells were effectively eradicated by the multifunctional Fe_3_O_4_ MNPs [122]. Furthermore, Tudisco and team fabricated a Fe_3_O_4_ MNPs functionalized with a phosphonic acid monolayer via a flexible synthetic approach that covalently binds to the surface of the gH625 peptide, a membranotropic peptide present in BBB and consequently promotes BBB permeability. According to findings, confocal laser scanning microscopy illustrated that the absorption of developed MNPs in immortalized human brain microvascular endothelial cells was more pronounced than folic acid-functionalized MNPs after 24 h. However, both functionalized systems demonstrated internalization in brain tumor cell lines. These results revealed a realistic approach for developing functional nanostructures that could cross BBB and then access specific tumor brain cells by improving the internalization of MNPs by endothelial cells [84].

A study by Costachi et al., used helianthin (He) as a carrier system for MNPs to treat cancer cells generated from GBM. Cell proliferation was assessed after cells had been exposed to various doses of HeNPs at 24, 48, and 72 h. According to the findings, GBM cells tolerated the treatment with NPs well, and their vitality was slightly enhanced. Furthermore, it was observed that human GBM cells were cytotoxic when exposed to He-loaded Fe_3_O_4_ MNPs [123]. Notably, ferritin-coated DOX nanocarriers that specifically bind to TfR also promote drug accumulation across BBB and targeted glioma delivery [85,124].

### Gold nanoparticles (AuNPs)

Nanoscale drug delivery systems are successfully transported to the brain through active targeting, which includes absorptive, receptor, and transporter-facilitated transcytosis. In addition, the tumor microenvironment, which includes factors like low pH, the EPR effect, and others, is crucial to the development of tumors and can be used to target brain tumors. Small, simple, and straightforward nanosystems are necessary to target brain malignancies efficiently. Given their versatility, gold nanoparticles (AuNPs) can be employed for drug administration, targeting, and diagnosis [125].

The BBB contains large amounts of LDLR, which overexpress in malignant glioma cells. In an infiltrative F98 glioma rat model, AuNPs functionalized with LDLR ligands (ApoB@AuNPs) sought to cure tumor microenvironment using transmitted proton sensitization. When compared to using only protons, proton sensitization had a therapeutic efficacy of 67%–75% higher in treating the tumor microenvironment and bulk tumor volume. According to immunohistochemistry, AuNPs significantly treat the proliferation of endothelial and migrating tumor cells of invasive microvessels in the tumor microenvironment while keeping normal tissues. Overall, findings suggest that using AuNPs functionalized with LDLR ligands is a viable approach for treating infiltrative cancerous glioma and preventing BBB passage [126]. Notably, Liu and associates presented a novel approach for the treatment of GBM using plasmonic gold nanostars as photothermal inducers for Synergistic Immuno Photothermal Nanotherapy (SYMPHONY), which coalesce immunotherapy with the use of checkpoint blockade and gold nanostar and laser-induced PTT. SYMPHONY successfully heralded the effective formation of immunologic memory in the CT-2A glioma cell line-mediated mouse flank tumor model and produced long-term survivors that resist re-exposure to cancer cells [127].

Wang et al., produced a TMZ-conjugated AuNPs functionalized with an anti-ephrin type-A receptor antibody as an intranasal delivery mechanism. The method can traverse the BBB and target active glioma cells while reducing peripheral toxicity and drug resistance, improving TMZ targeting for gliomas, and increasing treatment efficacy. Studies conducted *in vitro* found that anti-EphA3-TMZ@AuNPs dramatically increased cellular uptake and toxicity. When orthotopic glioma-bearing rats were used to test the anti-glioma effectiveness *in vivo*, the results showed that anti-EphA3-TMZ@AuNPs significantly boosted tumor-cell apoptosis compared to TMZ and extended the median survival period to 42 days, affirming it as an efficient intranasal drug delivery stratagem for the treatment of GBM [87]. Nevertheless, for plasmonic PTT, highly effective photosensitive nanosized particles are Keratin-coated AuNPs. According to experiments, ker-AuNPs have remarkable biocompatibility, superb cellular absorption, and localized photothermal heating properties. Based on their structural and functional characteristics, Ker-AuNPs have been recognized as a potential novel technique in the realm of biocompatible agents for plasmonic PTTs for cancer-associated disorders [128].

### Silver nanoparticles (AgNPs)

Various investigations have been carried out to design nanotechnology-based therapeutics and diagnostics agents. Liang and associates studied the outcomes of silver nanoparticles (AgNPs) on human glioma U251 cells and their function as an amalgamation with TMZ against glioma cells. AgNPs developed with 26 nm particle sizes confirm dose-determined cytotoxicity on U251 cells [90]. In another study, an evaluation of AgNPs on hypoxic glioma cells was conducted to measure the radiosensitizing efficacy. AgNPs demonstrated elevated capacity in radiosensitization in the hypoxic cells compared to normoxic cells [129]. AgNPs were also developed for neuroepithelial tumors, and results indicated that AgNPs significantly affect the proliferation and activation of the intrinsic apoptotic pathway of GBM cells cultured in an *in vivo* model [91].

Simsek and associates fabricated *Lavandula angustifolia* (LA) extract-loaded AgNPs and explored their anti-proliferative and apoptotic potential against the U87MG GBM cancer cell line. The developed NPs provoke a dose-determined decline in proliferation and augment cytotoxicity in U87MG cells. The results also proposed that La-AgNPs stimulate cell death in U87MG cells via p53-associated intrinsic apoptotic pathways [88]. In another investigation, *Kaempferia rotunda* tuberous rhizome extract-loaded AgCl-NPs were developed and evaluated for their anti-cancer activity against glioblastoma stem cells (GSCs) *in vitro* and Ehrlich Ascites Carcinoma (EAC) cells in mice. The fruit extract-loaded AgCl-NPs impeded 77.2% and 71% of GSCs growth at 32 µg/mL, corresponding IC_50_ values of 6.8 and 10.4 µg/mL. Additionally, they showed 32.3% and 55% inhibition of EAC cell growth *in vivo* at doses of 6 and 12 mg/kg/day, respectively, indicating its persuasive anticancer potential [89]. Indeed, radioresistance drops off the effectiveness of radiotherapy, eventually triggering tumor recurrence and spread. An innovative form of nano-radiosensitizer was designed using aptamer-AS1411 and verapamil (VRP) conjugated bovine serum albumin (BSA)-coated AgNPs (AgNPs@BSA-AS-VRP). They found that the developed nanocarriers significantly amplified the intracellular NPs’ level through AS1411-facilitated active targeting and reserve P-gp activity [130,131].

### Metal oxide nanoparticles

Scientists have fabricated and examined various nanomaterials, but those based on metal oxide NPs have captured their interest due to distinct features. Numerous biomedical applications, such as bioimaging, medication and gene delivery, antioxidant therapy, photodynamic therapy (PDT), dentistry, hyperthermia treatments, and wound healing, have all been investigated with metal oxide NPs [132]. Zinc oxide (ZnO) spherical NPs have recently drawn considerable focus in biomedical domains as an anti-tumor agent. In contrast, reports of the non-spherical ZnO NPs on tumor cell killing effectiveness are rare. Dang and associates prepared ZnO spiky NPs that were found to be more effective at eliminating tumor cells. To be more precise, they demonstrated a long-lasting cytotoxic outcome in killing tumor cells, as many spiky NPs remained on the surface of the plasma membrane of tumor cells even after repeated co-incubation [92]. Functionalized Ln_2_O_3_ NPs can be utilized as MRI contrast agents for imaging that targets tumors and other types of lesions, as well as for image-guided cancer therapy. Gd_2_O_3_ NPs have primarily been thoroughly studied as tumor-targeting T1 MRI contrast agents. T2 MRI is also achievable because Ln_2_O_3_ NPs have noticeable paramagnetic moments at ambient warmth. Due to their lofty X-ray attenuation power, Ln_2_O_3_ NPs are also suitable as contrast agents in X-ray computed tomography [133].

Conjugated polymer NPs (CPNPs) have recently arisen as sophisticated polymeric nanoplatforms in biomedical fields because of astonishing characteristics such as lofty fluorescence brightness, vast one and two-photon absorption coefficients, outstanding photostability, and colloidal steadiness in water and physiological solutions. Furthermore, the little cytotoxicity, simplicity in functionalization, and ability to adjust through dopant incorporation transform them into admirable theranostic agents with multipurpose functions, including imaging and treatment. Arias-Ramos et al., developed CPNPs by incorporating a metal oxide magnetic core into the matrix amid the nanoprecipitation approach. The resultant Fe_2_O_3_ NPs doped CPNPs had excellent cell addition depending on the exposure to the concentration of Fe_2_O_3_NPs and were biocompatible in the GBM cell line *in vitro*; nevertheless, including Fe_2_O_3_ into the CPNPs did not affect the PDT [134].

### Silica nanoparticles (SiNPs)

The most prevalent brain tumor is glioma and chemotherapy for glioma is restricted by inadequate availability and low BBB permeability. Indeed, mesoporous silica nanoparticles (SiNPs) recently flourished to show various applications involving controlled and targeted release by their tunable pore diameter and lofty surface area [94]. PTX was designed to be transported across BBB via the LDL receptor-related protein 1 overexpressing on glioma cells and BBB. The homogenous size distribution, more tremendous drug loading potential (11.1%), and prolonged release of Ang-2 modified lipid-coated mesoporous SiNPs of PTX (ANG-LP- SiNPs-PTX) are a few of its distinguishing features. *In vitro* tests showed that the developed formulation had a more substantial permeability and increased cellular apoptosis, which improved the targeting efficiency of PTX [93].

Erythrosine B (ERY) loaded biotin-functionalized SiNPs were also developed for PDT. The co-condensation process was used to produce amino SiNPs, which were then functionalized by amide linkage with biotin. The SiNPs and ERY were tightly bound together, preventing premature leaking into the bodily fluids. To demonstrate how the nanoplatforms produce 1O2, the well-known singlet oxygen scavenger function of uric acid was used. ERY/SiNP and ERY/SiNP-B samples have revealed prospective for PDT against GBM cancerous cells by destroying around half of T98G cells [94].

### Carbon nanotubes (CNTs)

It has become difficult to transport chemotherapeutics to the brain for the treatment of tumors; hence, significant research has been carried out to raise the likelihood of brain-associated drug delivery. Due to their tactical features in the transport of drugs to the target site, carbon nanotubes (CNTs) have arisen as promising systems among the numerous drug delivery technologies now in use. The use of CNTs in drug delivery methods provides a variety of ways to cross BBB, including the potential to extend drug circulation and allow functionalization with targeted molecules to expedite BBB transport. According to several research findings, CNTs can be a steady drug delivery method for targeting brain tumors [135]. Alizadeh and associates investigated that single-walled CNTs non-covalently functionalized with CpG (SWNT/CpG) that maintain immunostimulatory features, explicitly restrict the migration of glioma cells but not macrophages, while having no negative effects on cell viability and proliferation. SWNT/CpG also explicitly reduces the NF-β activation in glioma cells while triggering macrophages by inducing the Toll-like receptor 9 (TLR9)/NF-β pathway. Thus, SWNT/CpG is the first nanomaterial that, as far as it limits cancer cell migration while boosting the immune system [136]. Romano-Feinholz et al., also explored a possible therapy against neoplasms. They developed four different types of multiwalled CNTs (MWCNTs) to study the effect of the cell lysis and possible additive effect in combination with TMZ using astrocytes and RG2 glioma cells. The average diameter of both pristine undoped CNTs and pristine nitrogen-doped CNTs was 22 and 35 nm, respectively. Findings revealed that these CNTs can be used as adjuvant intervention and regular treatment to boost the overall survival in malignant glioma [96].

### Nanodiamonds (NDs)

Nanovehicle’s limited capacity to penetrate the BBB for tumor site is hampered by their short circulation time, poor BBB penetration, and less specific targeting. Consequently, it is challenging to make a definitive diagnosis of malignant brain tumors. Leung and associates studied how to image photostable biopolymer-coated nanodiamonds (NDs) inside the brain that can target tumors. The tumor vasculature-targeting tripeptides RGD and PEGylated denatured BSA were used to label NDs. The modified NDs in various buffer solutions demonstrated high colloidal stability. Additionally, discrete dcBSA-PEG-NDs were observed to penetrate the *in vitro* BBB model more successfully than aggregated NDs. Notably, after systemic injection, RGD-dcBSA-PEG-NDs may specifically target the tumor location in mice with the U-87MG, in contrast to non-targeting NDs. This discrete ND system, which effectively visualizes brain tumors while posing little risk to other vital organs, merits further research into its potential use as a cutting-edge platform for non-invasive theranostics at various phases of brain disorders in people [97].

Furthermore, Moscariello and associates examined NDs as a robust diagnosing platform for various biological uses by formulating targeted brain cells absorbed fluorescent NDs coated with a specially made human serum albumin-based biopolymer called dcHSA-PEG. Systematic administration of dcHSA-NDs in a mouse model supports their ability to traverse the BBB. It could track dcHSA-NDs down to single cell level, demonstrating their assimilation into astrocytes and neurons, thus providing the first evidence of systemic ND brain delivery and raising the possibility of direct cell-cell and BBB-crossing transport pathways [99]. Chen and associates investigated molecular insights into the DOX allied NDs (nano-DOX) in human GBM cells. The developed nanoformulation inhibited autophagy, facilitating apoptosis by blocking HMGB1 expression [98]. Also, in another study, it was discovered that nano-DOX impedes STAT3 activation in tumor cells and lowers IL-6 production in astrocytes, indicating that nano-DOX has the potential to break the STAT3/IL-6-mediated reciprocal activation loop among glioma cells and astrocytes [137].

Interestingly, Nikam and associates focused on developing and characterizing thiol-functionalized reduced graphene oxide (TrGO), an innovative proposal for the amide bonding-based loading of the anticancer medication methotrexate (TrGO-MTX). TrGO-MTX showed a considerable suppression rate in U-373 MG cell lines compared to pure MTX [138]. Maziukiewicz et al. developed conjugated NDs with biomimetic polydopamine and indocyanine green (ICG) for GBM PTT therapy. The results showed an improved therapeutic effect and more excellent elimination of glioblastoma cell*s* [100].

### Quantum dots

In recent years, there has been a growing interest in quantum dots across biomedical fields, primarily because of their remarkable optical and electronic properties. Specifically, graphene quantum dots (GQDs) have garnered attention in biomedical applications due to their excellent biocompatibility and the distinctive “molecule-like” qualities that set them apart from other types of quantum dots [101]. Sung et al. fabricated docetaxel-loaded GQD and results affirmed that a single irradiation treatment with GQDs effectively inhibits tumor growth within 21 days [139]. Also, Perini et al. showed that the surface chemistry of GQD amends the sensitivity of tumor cells to chemotherapeutics. On co-administration with DOX, they displayed a synergistic effect on tumor cells, arbitrated by the alteration in membrane permeability triggered by the surface of quantum dots [101]. Qiao and associates fabricated fluorescent carbon dots using a variety of d-glucose (Glu) to l-aspartic acid (Asp) molar ratios. According to the findings, carbon dots made with the ideal Glu/Asp molar ratio (7:3) had the best capacity to target C6 glioma cells. The research further demonstrated that the interaction between molecular design and associated functions creates a new avenue for cutting-edge NPs with biological applications [140].

Interestingly, Mansur and associates designed and characterized innovative hybrid nanostructures incorporating mitochondria-targeting pro-apoptotic peptide (KLA) with functionalized carboxymethylcellulose (CMC) and cysteine with fluorescent inorganic semiconductors. According to the findings, CMC-peptide macromolecules generated supramolecular vesicle-like nanostructures with aqueous colloidal stability suited as a nanocarrier for better cancer targeting via active and passive modes. The nanoconjugates’ optical and physio-chemical characteristics supported their applicability as a photoluminescent nanoprobe for intracellular tracking and bioimaging. The *in vitro* data also showed that the developed nanosystem had a substantial killing ability against GBM cells [102].

### Calcium phosphate nanoparticles (CaP-NPs)

Mitrach and associates developed calcium phosphate nanoparticles (CaP-NPs) utilizing a novel terpolymer for transferring siRNA to brain cancer cells. Importantly, siRNA suppression of survivin in F98 rat brain cancer cells was observed because of highly efficient siRNA binding to the CaP-NPs. Organotypic brain slice cultures showed no adverse effects, while cytotoxicity tests using a conventional cell line produced minimal and temporary impact; however, more intrinsic research is still needed [141].

## Miscellaneous Nanocarriers

### Nanorobots

Cancer control has so far been one of nanotechnology’s notable beneficiaries. Nanostructure, robotics, healthcare, and computer system improvements lead to the development of nanorobots. Indeed, a nanorobot is a machine with a size between 0.1 and 10 µm, making it comparable to an RBC. This sophisticated robot may patrol the circulation, identify its intended victim, and then unleash a small but lethal load of drugs or NPs to eradicate cancerous cells. These nanoscale devices are helpful for the early detection, prognosis, and therapy of several diseases, including cancer [142]. Although these structures have unique potential, using inorganic materials can impact their functionality and lead to health problems in the body. To get around this, materials derived from nature are included in developing biomimetic nanorobots, which may effectively functionalize and overcome immunological reactions and lessen side effects. These biomimetic nanorobots may expand the possibilities for cancer imaging and treatment [143].

### Natural substrates as nanocarriers

Serum protein, a naturally occurring biomacromolecule, has recently become a flexible drug delivery system for diagnostic and therapeutic purposes in cancer nanomedicine. It has advanced biocompatibility, enhanced pharmacokinetics, and better targeting. Several serum proteins have been used for drug delivery. Due to their precise biological design and prospective therapeutic applications, serum protein-based NPs have drawn considerable interest, as demonstrated by the accomplishment of Abraxane^TM^ (PTX-bound albumin NPs). They succinctly outline the existing difficulties that must be overcome for serum protein-based nanomedicines to have a promising future [144].

## Clinical Translation of Nanomedicines

**Figure 4:**
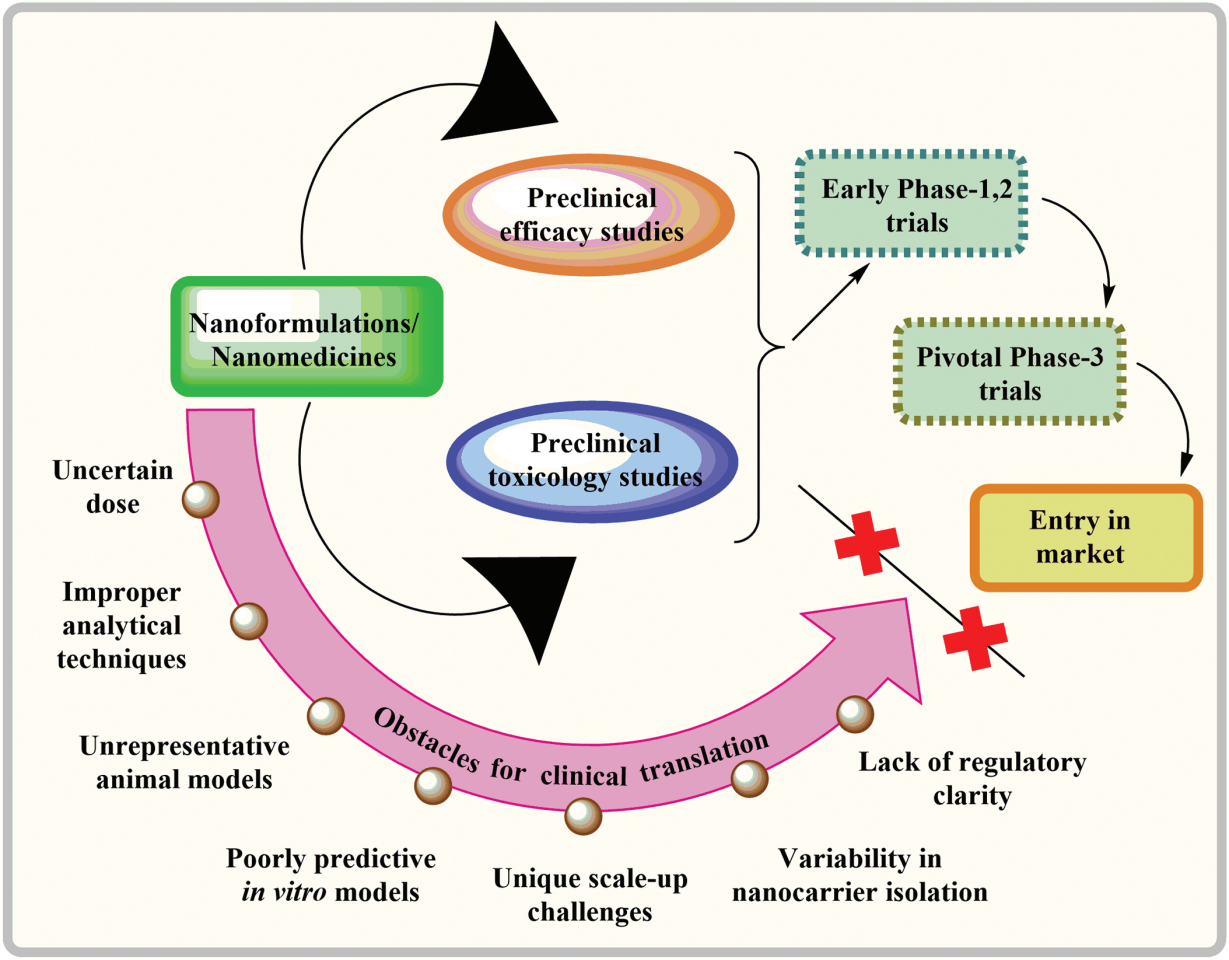
Barriers hindering the clinical adoption of nanomedicines for delivering drugs to the brain.

To ensure the successful clinical translation of nanotechnology-based treatments, they must be safe and offer significant benefits compared to the current standard of care. As of 2022, there were limited commercially accessible cancer treatments utilizing nanotechnology. The majority of these treatments were modifications of traditional chemotherapy drugs, such as DOX, PTX, and daunorubicin, encapsulated in liposomal formulations [145]. Nevertheless, developing nanomedicines for effectively transporting drugs to the brain for clinical applications is beset with numerous challenges, as illustrated in Fig. 4. Transitioning a research or publication-grade nanocarrier technology to a commercial product from clinical trials is not straightforward. Ideally, clinical studies may require up to five years, with full clinical implementation taking a decade [146,147]. Numerous studies have employed animal models like xenografts or flawed *in vitro* models such as brain endothelial cell monolayers to assess the anti-cancer efficacy of NPs. However, these models fail to faithfully replicate the intricacies of the human brain, thereby creating a significant disparity in the translation of nanoformulations to clinical applications. Moreover, accurately measuring the dispersion of nanocarriers and their uptake by the brain presents significant hurdles in clinical research [145,146]. In addition, varying methods of liposome synthesis can yield diverse end products akin to the challenges encountered in exosome isolation. Scaling up from the laboratory bench to pre-clinical to clinical production brings up additional challenges for nanocarriers. One of the most concerning issues is the lack of regulatory clarity, following ambiguous standards for assessing batch consistency and bioequivalence [145,147].

The first therapies for brain tumors are predicted to be more straightforward methods combining licensed drugs like liposomal DOX or TMZ with targeted ultrasonography or liposomal nanocarriers. Due to the highly unpredictable nature of GBM and the lack of effective treatment options for patients, regulatory agencies tend to be more flexible in their approval processes [148,149]. For example, the US Food and Drug Administration approved TMZ based on a minimal increase in survival from 12 to 14.6 months [150]. Bevacizumab was permitted to be used for its treatment even though it only provides symptomatic relief and has no impact on survival time [151]. Table 1 highlights some crucial pre-clinical studies done in the previous five years. Table 2 highlights clinical research that has been reported to date in the nanocarrier-based brain tumor therapy field.

**Table 1:** Nanocarriers developed in the previous five years for the treatment of GBM

Type of nanocarrier	Encapsulated drug	Method of preparation	Particle size	Cell line used	Dose	Inference/results	References
Liposomes	Docetaxel	Film hydration method	155–159 nm	U87-MG and hCMEC/D3	1 mg/kg	Liposomes significantly penetrate tumor spheroids and impede their growth Liposomes protract survival time	[78]
	——	Thin layer evaporation method	151 nm	U87-MG, bEnd.3, and C6	30 μg/ml	Liposomes improve BBB permeability Liposomes impede tumor growth by 94.2%	[79]
					1 mg/kg		
	Resveratrol	Thin film hydration method	211.2 nm	U87-MG	12.5–200 μM and 10 mg/kg	Liposomes prolong drug release Liposomes trigger apoptosis and inhibit cell-cycle Liposomes impeded tumor growth	[48]
Nanocapsule	Simvastatin	——	66 nm	U138-MG and C6	0.01–20 µM	Nanocapsules trim down tumor volume by 78%	[80]
					60 µg/mg		
Tf-modified NPs	TMZ and bromodomain inhibitor (JQ1)	Thin film hydration method	137 nm	U87-MG and GL261	TMZ: 0 to 1.25 mM and 2 mg/kg	NPs easily penetrate BBB NPs trigger DNA damage and cellular apoptosis NPs reduce tumors by 2 folds and augments survival	[81]
					JQ1: 0 to 1.6 μM and 2 mg/kg		
Dendrimers	BLZ945	——	5.3 nm	BV2 murine microglia	100 mg/kg	Dendrimers improve target delivery Dendrimers reduce tumor progression Dendrimers promote overall survival	[82]
	DOX	**——**	26.07 nm	U87-MG	20 μM	Dendrimers promote BBB infiltration Dendrimers improve the anti-tumor effect	[83]
					5 mg/kg		
MNPs	——	Alkaline co-precipitation	104 nm	A-172 and HBMEC	10–20 μg/ml	gH625@MNP upholds endothelial cell internalization	[84]
Magneto-Ferritin NPs	DOX	——	——	U87-MG	1 µM and 1 mg/kg	NPs exhibited controlled and targeted drug release NPs penetrate BBB easily	[85]
AuNPs	TMZ	——	45.88 nm	T98G	——	Intranasal administration of AuNPs prolongs survival rate by 1.64-fold AuNPs augment apoptosis	[86]
	TMZ	Citrate reduction method	46.12 nm	C6 and T98G	2.5−60 μM	AuNPs augment cellular uptake by 54.9% AuNPs elevate cell death by 14.1% AuNPs delay survival rate up to 42 days	[87]
AgNPs	*Lavandula angustifolia* extract	Green synthesis technique	≥100 nm	U87-MG	0–20 µg/ml	AgNPs diminish cell proliferation and trigger cell death via the p53-mediated intrinsic apoptotic pathway	[88]
	*Kaempferia rotunda*	——	17 nm	GSC	2–32 μg/ml	Down-regulation of STAT3, EGFR, PARP, and NOTCH2 gene expression Upregulation of p21, TLR9, NFκB, IL1, and TNFα	[89]
					6 and 12 mg/kg for 5 days		
	TMZ	——	26 nm	U-251	31 μmol/l	AgNPs trigger cellular apoptosis and arrest the G2/M phase of the cell cycle AgNPs improve TMZ sensitivity for GBM	[90]
	——	Electric non-explosive patented method	70 nm	In-ovo model	40 μg/ml	AgNPs impede cellular proliferation	[91]
ZnO spiky NPs	——	Precipitation method	45 nm	A-172	30–90 µg/ml	NPs trigger apoptosis NPs demonstrated anti-oxidative behavior	[92]
ANG-LP-SiNPs-PTX	PTX	——	106.37 nm	C6 and HBMEC	0.1–100 µg/ml	NPs elevate drug penetration by 10.74% NPs arrest the G2/M phase by 52.56% NPs improve survival time to 26.83 days	[93]
Biotin-functionalized SiNPs	Erythrosine B	Co-condensation technique	120 nm	T98G	1 µmol/ml	NPs significantly dwindle GBM tumor cell viability	[94]
Tf-modified porous SiNPs	DOX	Electrochemical anodization technique	167 nm	U87-MG and hCMEC/D3	5 μg/ml	Silica NPs release DOX in a pH-responsive manner NPs considerably infiltrated BBB NPs reduce cell viability by 2 folds	[95]
CNTs	TMZ	Pyrolyzation technique	22 to 35 nm	RG2	50 μg/ml	MWCNTs elevate apoptosis MWCNTs improve overall survival	[96]
NDs	——	——	69.35 nm	U87-MG cell	25–1000 μg/ml	NDs augment BBB permeability NDs effectively target brain tumors *in vivo*	[97]
	DOX	——	83.9 nm	U87-MG	2 μg/ml	NDs improve cellular autophagy that triggers apoptosis	[98]
	——	——	40–55 nm	bEnd.3	3 g/ml	NDs enhance BBB permeability and cellular uptake	[99]
	Biomimetic polydopamine	——	357.57 nm	U373-MG	——	NDs improve PTT by 10-fold	[100]
GQD	DOX	——	≥10 nm	U87-MG	50–250/ml	GQDs elicit synergistic outcomes on tumor cells and improve BBB permeability	[101]
Quantum dots	DOX	——	2.7 nm	U87MG	0.01–5 μM	Quantum dots diminish tumor progression by 41%	[102]
Bilirubin NPs	Cediranib	——	112.3 nm	C6 and HUVE	0.18 μg/mL and 3.6 mg/kg	NPs promote cellular apoptosis NPs significantly augment survival time to 231%	[103]
	PTX	——	117.8 nm		0.0854 μg/mL and 1.7 mg/kg		

**Table 2:** Current clinical trials assessing the therapeutic benefit of NPs against GBM

Intervention/treatment	Type of nanocarrier	Phase	No. of patients enrolled	Status	CT Identifier
AGuIX with stereotactic radiation	Gadolinium NPs	II	134	Recruiting	NCT04899908
Nanoliposomal Irinotecan (Onivyde) +TMZ	Liposomes	I	12	Terminated	NCT03119064
AGuIX with whole brain radiation therapy	Gadolinium NPs	I	15	Completed	NCT02820454
AGuIX with whole brain radiation therapy	Gadolinium NPs	II	100	Recruiting	NCT03818386
DOX loaded Anti EGFR Immunoliposomes	Liposomes	I	9	Completed	NCT03603379
NU-0129	AuNPs	Early I	8	Completed	NCT03020017

### Symptoms, monitoring, and prevention of nanotoxicity

The detrimental effects of NPs on human health have primarily been studied in animal models, referred to as “nanotoxic” consequences. Numerous organs may be affected by NPs toxicity, which is primarily brought on by cell membrane breakdown, cellular redox disruption, and following activation of reactive oxygen species (ROS) [25,152]. *In vitro* investigations, ROS assays, and electron microscopy of cell membranes are being employed extensively, but none are sufficient for evaluating clinical nanotoxicity. This is an essential vista for treating brain diseases because changes in neuronal membrane integrity can lead to incorrect action potential, cell death, edema, and many others. Final product testing usually adheres to the National Pharmacopeia of each Nation, which outlines the standards and test methodology for active components in a particular dosage form [153].

## Future Prospective

A significant challenge in the development of drug delivery systems in the brain has been demonstrated by decades of unsuccessful scientific research. Despite significant improvements in the majority of other tumors, GBM survival rates remain persistently poor. Nanomedicines have shown substantial promise in significantly improving outcomes for brain tumor patients. While preclinical animal experiments indicate the potential of nanomedicine in treating brain cancers, clinical trial data has not met expectations. The reasons for this disparity are not fully understood, but factors like the limited EPR effect and the diverse nature of human brain tumors are believed to contribute. There is optimism for improvement, aiming for nano-theranostics that meet criteria like reduced toxicity, increased efficacy, and prolonged treatment for brain cancers, moving closer to clinical success in neuro-oncology. The biosafety of nanomaterials remains a significant challenge for clinical translation. Appropriate criteria and effective analytical methods for nanoformulations-based products must be developed to carry out their translation process.

Future investigations should prioritize enhancing the development, fabrication, and characterization technologies to optimize therapeutic outcomes while minimizing cancer resistance and recurrence. Collectively, researchers are making strides to treat and improve the life expectancy of patients dealing with this devastating disease. Increased knowledge of GBM molecular pathways related to drug resistance, coupled with advancements in delivery strategies, can optimize nanomedicine for more effective GBM treatment. Despite challenges, optimism remains regarding the future of this field, believing that nanomedicine will revolutionize GBM therapies.

## Conclusion

A dreadful percentage of lives were lost due to cancer each year. The advancement of drug delivery systems for brain treatment has faced substantial obstacles despite extensive research efforts. Although progress has been made in treating various tumors, survival rates for GBM remain persistently low.

Researchers worldwide actively explore novel formulations to enhance brain drug delivery in this rapidly evolving field. Many challenges persist, especially in generating highly efficient, therapeutically beneficial, and practical treatments for this lethal disease. Existing research mainly suggests methods that subtly but notably improve brain drug administration. The employment of cutting-edge nanotechnology approaches to boost efficiency and reduce limitations has raised high hopes. With the evolution of nanoscience, nanoformulations help in accurate and quick diagnosis and targeted drug deliverance of therapeutic agents for brain tumor treatment. Nanoformulations provide high specificity, sensitivity and multiplexed measurement potential. Cellular toxicity and medication off-targeting adverse effects can also be decreased. Other salient characteristics that nanoemulsion appears to offer for nanotherapy is a wide surface area that permits a larger drug loading to be delivered to the specific tumor site. Novel advancements in nanoformulations will provide inventions that symbolize a standard change in the brain tumor treatment that can ominously contribute to better patient outcomes.

However, most of these technologies have yet to undergo rigorous clinical testing. The criteria for defining and quantifying nano-toxicity are also not precisely defined. Regulatory uncertainties impede advancements in delivering nanomedicines to clinical use. Current nanotechnology-based delivery methods have considerably improved BBB permeability and the accumulation of therapeutic agents in GBM while reducing systemic toxicity.

Multiple approaches are critically essential to comprehensively characterize nanoformulations, understand their properties, and validate their effectiveness in diverse preclinical scenarios. It can be concluded that new alternative treatments for brain cancer, as well as better formulations to defeat its therapeutic limitations, are still being investigated. Recent investigations in this arena are also very vital and can provide new perspectives.

## Data Availability

Not applicable.

## Abbreviations

ABC: ATP-binding cassette
AgNPs: Silver nanoparticles
AuNPs: Gold nanoparticles
BBB: Blood-brain barrier
BBTB: Blood-brain–tumor barrier
BDNF: Brain-derived neurotrophic factor
BECs: Brain endothelial cells
BSA: Bovine serum albumin
CaP-NPs: Calcium phosphate nanoparticles
CNTs: Carbon nanotubes
CPNPs: Conjugated polymer NPs
DOX: Doxorubicin
EGFR: Endothelial growth factor receptor
EPR: Enhanced permeability and retention
EVs: Extracellular vesicles
GBM: Glioblastoma multiforme
GFR: Growth factor receptor
GLUT: Glucose transporter
GQD: Graphene quantum dots
IDH: Isocitrate dehydrogenase
IL: Interleukin
LDLR: Low-density lipoprotein receptors
MNPs: Magnetic nanoparticles
MRI: Magnetic resonance imaging
MTX: Methotrexate
NDs: Nanodiamonds
NPs: Nanoparticles
PAMAM: Polyamidoamine
PDT: Photodynamic therapy
PEG: Polyethylene glycol
PLL: Poly-L-lysine
PPI: Poly-Propyleneimine
PTT: Photothermal therapy
PTX: Paclitaxel
RMCT: Receptor-mediated cell transfer
ROS: Reactive oxygen species
SiNPs: Silica nanoparticles
SLC: Solute carrier
TAMs: Tumor-associated macrophages
TfR: Transferrin receptor
TLR9: Toll-like receptor 9
TMT: Transporter-mediated cell transfer
TMZ: Temozolomide
TTF: Tumor therapeutic field
VEGF: Vascular endothelial growth factor
VRP: Verapamil
ZO-1: Zonulachludens-1
